# The Beginner Laparoscopists Trends in the Learning Process of Laparoscopy for Adnexal Gynecological Pathologies—The Experience of Our Center

**DOI:** 10.3390/healthcare11121752

**Published:** 2023-06-14

**Authors:** Romina-Marina Sima, Anca-Violeta Crăițan, Liana Pleș, Florin Bobircă, Mihaela Amza, Gabriel-Petre Gorecki, Mihai-Teodor Georgescu, Bashar Haj Hamoud

**Affiliations:** 1 Department of Obstetrics and Gynecology, “Carol Davila” University of Medicine and Pharmacy, 020021 Bucharest, Romania; romina.sima@umfcd.ro (R.-M.S.); anca-violeta.craitan@rez.umfcd.ro (A.-V.C.); liana.ples@umfcd.ro (L.P.); 2 The “Bucur” Maternity, “Saint John” Hospital, 040294 Bucharest, Romania; 3 Department of Surgery, “Carol Davila” University of Medicine and Pharmacy, 050474 Bucharest, Romania; 4 Surgery Department, Dr. Ion Cantacuzino Clinical Hospital, 011437 Bucharest, Romania; 5 Faculty of Medicine, Titu Maiorescu University, 040441 Bucharest, Romania; gabriel.gorecki@doc.utm.ro; 6 Department of Anesthesia and Intensive Care, CF2 Clinical Hospital, 011464 Bucharest, Romania; 7 “Prof. Dr. Al. Trestioreanu” Oncology Discipline, Carol Davila University of Medicine and Pharmacy, 252 Fundeni St., 050474 Bucharest, Romania; mihai.georgescu@umfcd.ro; 8 Department for Gynecology, Obstetrics and Reproductive Medicine, Saarland University Hospital, KirrbergerStraße 100, Building 9, 66421 Homburg, Germany; bashar.hajhamoud@uks.eu

**Keywords:** learning process, laparoscopic gynecology, gynecological surgery, laparoscopy, ovarian cyst

## Abstract

Background: Laparoscopy for benign ovarian pathology is the appropriate surgical approach and it has many well-known advantages. Minimal invasive gynecological surgery increases the quality of life of the patient. The learning process of laparoscopy is difficult and requires many interventions to acquire manual skills. The objectives of the study were to assess the learning process of laparoscopy for adnexal pathology surgery performed by beginner laparoscopists. Materials and Methods: This study included three gynecological surgeons who were beginners in laparoscopy and who were named A, B, and C. We collected information about patients, diagnosis, surgical technique, and complications. Results: We have analyzed the data from 159 patients. The most frequent primary diagnosis was functional ovarian cyst, and the laparoscopic cystectomy was performed in 49.1% of interventions. The need to convert a laparoscopy into laparotomy was necessary in 1.3% of patients. There were no cases of reintervention, blood transfusion, or ureteral lesions. The duration of the surgical intervention varied statistically significantly according to patient’s BMI and to the surgeon. After 20 laparoscopic interventions, a significant improvement was found in the time needed to perform ovarian cystectomy (operators A and B) and salpingectomy (operator C). Conclusions: The process of learning laparoscopy is laborious and difficult. We found a significant decrease in operating time after a twenty laparoscopic interventions.

## 1. Introduction

The first laparoscopy was performed in 1901 on a dog by Georg Kelling during a scientific meeting, and he called this intervention “coelioscopy” [1,2]. In 1910 a Swedish doctor performed “laparothoracoscopy,” the first endoscopy of the human abdominal cavity and the chest [3]. The first laparoscopy made in the USA was named “organoscopy” and it was performed in 1911 [4]. The development of laparoscopy required many steps; the greatest progress was made between 1960–1980. A transition of this technique from a diagnostic to a therapeutic purpose was observed. A gynecologist performed the first laparoscopic appendectomy in 1980, in Germany. The rapid development of laparoscopy had an important impact on gynecological surgery [5]. A reference moment in the development of laparoscopy in the treatment of gynecological diseases was represented by the first bowel resection in the case of a patient with endometriosis in 1988 by Camran Nezahat [6]. Important progress for gynecological laparoscopy was observed from 1989 to 1992. The laparoscopic hysterectomy technique was developed and improved by Camran Nezhat, Harry Reich, S. Kovac, and G. Magi [7].

Over the years, laparoscopy has been preferred over classical surgical techniques due to the advantages of minimally invasive techniques. It is currently used worldwide in a variety of surgical diseases due to lower operating time, fewer hospitalization days, diminished intraoperative hemorrhage, and cosmetic skin incisions [8,9]. That is why it is necessary to analyze and standardize the process of learning this type of surgery by evaluating parameters that characterize the surgical act and by using this information to build learning curves [10]. As a result, young doctors are able to learn the method more efficiently.

Currently, laparoscopy is the gold standard for surgical interventions. The perceptual obstacles, the stressful environment in the operating room, and the high costs of these types of interventions represented a challenge for the process of learning the basic techniques in laparoscopy. Thus, it was necessary to adopt new educational models. Medical simulation and virtual reality training have been developed with very good results in the learning process [11].

Each learning process consists of two distinct phases: an initial phase with an increased learning rate, along with a prolonged surgery time and a higher complication rate, and a second phase, with reduction of both procedure duration and complication rate [12,13,14]. Consequently, a graphic representation of the learning process has a sinusoidal aspect, initially ascending and followed by a descending phase after the reach of the plateau level that marks the end of the learning process. The learning process for less invasive procedures is considered longer and more difficult in comparison to the one for open surgical procedures, but it has been shown that when inexperienced subjects received equal training in these two types of techniques, the overall skills obtained were similar by both methods [15]. Moreover, it has been postulated that the rate of complications is decreasing significantly when the volume of surgical workload is getting higher overtime [16].

The Royal College of Obstetricians and Gynecologists (RCOG) published a guide in which laparoscopic gynecological interventions were divided according to the degree of difficulty into three levels. Level 1 included diagnostic laparoscopy, tubal patency tests, and sterilization. Level 2 included laparoscopic interventions at the level of the adnexal such as cystectomy, adnexectomy, surgical treatment of ectopic pregnancies, and subtotal hysterectomy. Difficulty Level 3 included myomectomy, total hysterectomy, and surgical treatment of endometriosis, urinary incontinence, or pelvic floor disorders. It was found that 73% of surgeons performed basic laparoscopic surgeries. Frequently, the explanation was the fact that interventions with a high level of difficulty require a longer operative time and long-term learning [17].

## 2. Materials and Methods

The aims of this study were to evaluate the learning process of laparoscopic surgery for adnexal benign gynecological pathology and to observe the direction of evolution of this minimally invasive surgery.

This study was performed between November 2019 and June 2020 by retrospectively analyzing clinical data from patients admitted to Bucur Maternity, Bucharest from 4 January 2015to 19 December 2018. Clinical data were collected anonymously from both observation charts and clinical records of the patients attending the study. Those patients were operated on laparoscopically by three gynecologists from Bucur Maternity whose names were concealed due to confidentiality reasons and replaced with the letters A, B, and C. An important observation is that the three operators had similar previous experiences before this study with a medium of approximately 30 laparoscopies for benign gynecological conditions. The operation time was noted from when the patient entered the operating room until the patient awaked from general anesthesia.

This study included all laparoscopic surgeries that were performed for a benign adnexal pathology. The histopathological reports did not indicate the presence of malignant tumors in the 159 cases. We collected information about patients, diagnoses, surgical techniques, and complications.

The data collected were analyzed using the SPSS Statistic software, version 23 (Armonk, NY, USA). We used descriptive statistics for mean, standard deviation, median, and frequencies. When we wanted to evaluate the relationship between two parameters, we used the independent sample *t*-test and one-way ANOVA. If the *p*-value was less than 0.05, then the correlation was considered statistically significant. Bar charts and scatter plots were created with data.

The limitations of this study were represented by the relatively reduced number of patients included in the study, the small number of surgeons practicing laparoscopy in our hospital, and the low frequency of complex ovarian pathologies. 

## 3. Results

Our study included a total of 159 interventions performed by three operators. 

The age of the patients varied between 19 and 56 years, with an average of 33.28 ± 7.80 years. Most surgical interventions were performed on patients under 35 years (63.3%). The body mass index (BMI) had a mean value of 26.12 ± 3.26 kg/m^2^ with a variation between 19.40 and 37.10 kg/m^2^.

According to the diagnoses, we found that 64.8% of the cases represented an ovarian pathology while 35.2% had a tubal pathology. The most common diagnoses were functional ovarian cyst (25.8%), ectopic pregnancy (22.0%), endometriotic ovarian cyst (10.7%), adnexal tumor (8.2%), and dermoid ovarian cyst (6.9%). Numerous secondary diagnoses were identified. Pelvic adhesion syndrome was present in the case of 39 patients (24.5%), while hemoperitoneum was present in 23 patients (14.5%). A less common condition such as Fitz-Hugh-Curtis syndrome was found in two cases (1.2%).

We analyzed different technical aspects of laparoscopic surgery. We found that for pneumoperitoneum the Veress method was the most-used laparoscopic approach (59.7%), followed by the Hasson method (40.3%). Carbon dioxide pressure varied between 11 and 13 mmHg; in 141 cases a pressure of 12 mmHg was maintained (88.7%). The number of trocars used was between two and five, in 57.9% of cases three trocars were used, while 40.3% of cases required the use of four trocars.

Ovarian cystectomy was the most common type of surgical intervention performed (49.1%). Other procedures completed were partial salpingectomy (31.9%), unilateral adnexectomy (13.2%), ovariectomy (4.4%), and adhesiolysis (28.9%). Tubal permeability testing with methylene blue was performed in 5.1% of cases. 

The duration of the surgical interventions varied between 50 and 180 min, with an average duration of 113.26 ± 27.28 min. The patients were hospitalized for a variable number of days, between three and six days. Most patients were hospitalized for four days (60.4%). Peritoneal drainage was used in 100% of cases and in 1.9% of cases two tubes were used for peritoneal drainage. The conversion of the laparoscopic intervention into laparotomy intervention was necessary in two cases (1.3%). There were no ureter injuries, reinterventions, or blood transfusions.

We used the independent sample *t*-test and one-way ANOVA to analyze the relationship between the duration of the surgery and the pathology, the operator, the type of laparoscopic approach, the number of exchanges used, and the days of hospitalization (Table 1). In Table 1, we looked at things as a whole in terms of laparoscopy interventions for benign adnexal tumor pathology. In this table we did not take operation types into account. We identified that the duration of surgery did not have a statistically significant correlation with any of these variables. Despite all this, we noticed that the duration of surgery was significantly correlated with the body mass index of the patients (*p* = 0.001); if the BMI increased, then the duration of the surgery also increased. The type of procedure performed significantly influenced neither the duration of the surgical intervention nor the number of trocars used.

The two surgery conversions into laparotomy interventions were performed by different operators, A and C. We analyzed these two cases and concluded that endometriotic ovarian cyst represented the main diagnosis for the conversion case of surgeon A and it was intervention number 27 out of 47 in chronological order. For operator C the diagnosis for the conversion case was dermoid cyst, representing in chronological order interventions for 43 out of a total of 53. 

We chose to evaluate the parameters for two of the most important gynecological surgeries: ovarian cystectomy and unilateral adnexectomy (Table 2). We found that there was a statistically significant difference between the average time needed to perform the ovarian cystectomy between operations A and C (*p*< 0.05). The two complications that were reported in Table 2 are represented by the conversions into laparotomy due to the adhesion syndrome that changed the anatomy of the pelvis and did not allow the operator to continue the surgeries safely. These two patients had had previous abdominal surgeries. 

**Figure 1:**
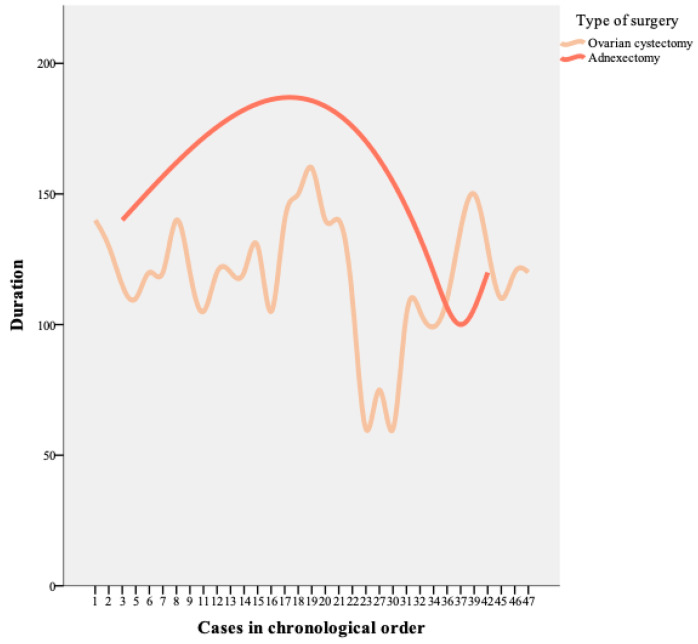
Operator A.

The evolution over time of the duration of surgeries was analyzed. We noticed that for gynecologic surgeon A there was an improvement in the operating time for the adnexectomy (Figure 1).

We divided the total number of surgeries for each gynecologist into two phases. In this division, the type of surgery performed was not considered and it was established that each case brought improvements in the technique and surgical skills of the operator.

Thus, the first phase was represented by the first 20 laparoscopic surgeries; the second phase was represented by the rest of the surgeries for each operator. 

We compared the duration of ovarian cystectomy and total or partial salpingectomy in the two phases using an independent samples *t*-test. We observed that there was a statistically significant improvement in terms of operating time for ovarian cystectomy for surgeons A and B (Table 3). In our study, a relatively small number of cases was included, which after the analysis did not result in more than five cases of ovarian cyst type for each operator in each learning phase. There was no difference in the ratio of diseases, including endometriotic ovarian cyst, dermoid cyst, functional cyst, and so on, between phases I and II. Operator C had a significant improvement in performing salpingectomy (Table 4). 

**Figure 2:**
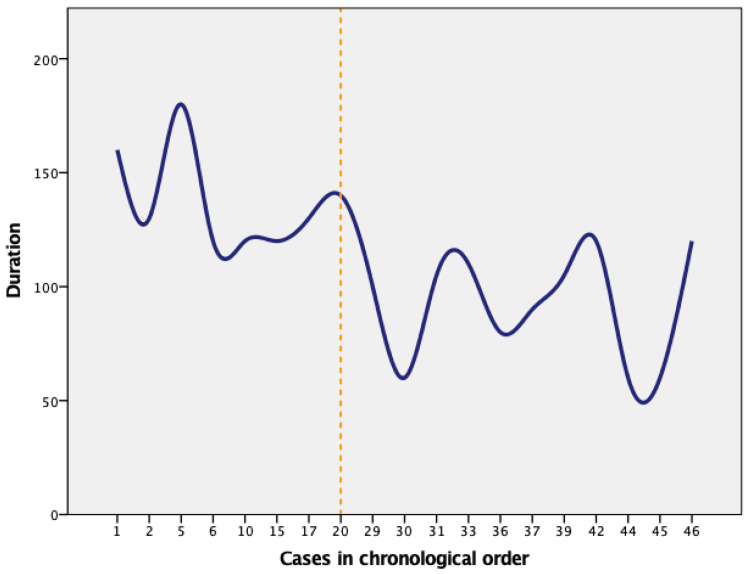
Laparoscopic salpingectomy performed by operator C.

The evolution of performing salpingectomy by gynecologist C was represented in Figure 2. The vertical line in Figure 2 indicated laparoscopy number 20 from the total number of surgeries performed by operator C, which marked the limit between the two learning phases.

## 4. Discussion

Laparoscopic surgery, in contrast to laparotomy, had demonstrated numerous advantages during the past 30 years, as it is a less invasive procedure and presents faster recovery time, shorter hospitalization, fewer infections, and less pain [8,9]. Our data showed that the mean duration of laparoscopic procedures was about 113 min and most patients required four days of hospitalization after surgery. A decrease in perioperative complications and in mean operating time has been often used to evaluate the learning process [18,19,20,21]. 

In our study, the most common gynecological pathology was represented by the ovarian cyst. A study conducted in a hospital from Poland analyzed 326 fertile women who needed a surgery on the ovarian tumor and concluded that unilateral benign cysts were the most frequent pathology. Furthermore, few patients had a malignant tumor, but it was usually in an early stage of the disease [22]. It was also observed in a prospective study that laparoscopy was safe and feasible for patients with large benign ovarian cysts [23].

The rate of intraoperative and postoperative complications was reduced, which can be explained through the young patients, the normal or slightly raised BMI, or the professionalism of the operating team. Leon Morgenstern highlighted in his publication the risks of the learning curve, the morbidity, or even the mortality rate caused by this learning process, and he considered the possibility of complications due to it not being reported often enough [24]. Hopkins reported in his publication an increased risk for ureteral injury and vesical-vaginal fistula in the early stages of learning laparoscopy in gynecologic procedures [25].

In our study there was no case which required reintervention. Wattiez et al. conducted a study on 1647 cases to evaluate the possibility of introducing the laparoscopic technique in the treatment of the benign uterine pathology, and the results revealed a reduced necessity of reintervention in the second phase of the study. Moreover, conversion to laparotomy was needed more frequently in the first phase of the study due to ureteral lesions, and this need was diminished in the second phase, where conversion to laparotomy was needed only in complicated cases [26]. The conversion to laparotomy was needed in less than 1.3% of interventions, especially in older patients, in patients with a higher BMI, or in patients with conditions such as extrauterine pregnancy and pelvic adhesions. The rate for conversion to laparotomy varied in the literature from 0.03% to 6.6% [9,27,28]. The conversion of the procedure generally occurs in the early learning phase, but sometimes there are situations which require changing a laparoscopy into a laparotomy, even for surgeons who completed the learning curve. Garett et al. mentioned in their study the necessity for conversion in six of eight patients due to broad adhesions and advanced diseases [29].

The results of this study showed no ureteral lesions, but there are other studies that reported such injuries. Wattiez et al. mentioned an incidence of 0.6% for ureteral injuries during the early process of learning for hysterectomy and a lower incidence (0.2%) during the second period of learning (managed intraoperatively, without the need for conversion [26]. They noted as risk factors for ureteral injuries excessive bleeding, very large uterus, and endometriosis [26]. Another retrospective analysis performed on 1706 patients after a laparoscopic supracervical hysterectomy described a total of five intraoperative cases (0.3%) complicated with bladder injury, ureter injury, or severe bleeding [28]. No patients registered in our study received blood transfusions, although they may be necessary in some circumstances. It is well-known in the literature that bleeding is one of the most major complications and it is a main reason for conversion to laparotomy [26].

In the learning process for laparoscopic hysterectomy (LH), Twijnstra et al. concluded that 22–25 LHs should be performed for reaching the plateau [30]. Similarly, Garry et al. suggested that after 25 cases the learning curve is completed [31]. There has also been a study published in 2016 by Terzi H et al. that analyzed the surgical learning process in benign pathology which detected a significant reduction in operating time between cases 50–100, without an additional decrease after this group. They also concluded that the plateau occurred during cases 71–80 [32]. A similar tendency was observed in another study that evaluated the learning curve of laparoscopic hysterectomy associated with removal of pelvic and paraaortic lymph nodes in patients with cervical cancer [33]. A study that included 576 laparoscopic hysterectomies concluded that the learning process of this type of surgery was safe for the patient, and an improvement in operative time was observed after 100 interventions but without reaching a plateau [34]. The most challenging moment in laparoscopic hysterectomy is when the uterus is excised; the incision is made at the level of the vagina and then the vaginal cuff is sutured. This step requires a long period of training to prevent complications [35].

Regarding the surgical treatment of pelvic organ prolapse, it was observed that in the case of laparoscopic sacrocolpopexy, the operating time decreased after 30 interventions, and an adequate operative performance appears after 60 performed procedures. It was found that in the case of laparoscopic pectopexy, the operating time stabilized after 28 interventions. The CUSUM analysis showed that 38–40 interventions of this type are necessary to master the surgical technique [36].

Laparoscopy is a safe and feasible technique in the case of malignant gynecological pathologies. The increase in the experience of the surgeon was accompanied by a decrease in the amount of blood lost intraoperatively, but it was found that it did not influence the number of excised lymph nodes or the number of days of postoperative hospitalization [37].

We found that after a total of 20 laparoscopic interventions, all three operators included in the study presented a significant decrease in operating time for different types of surgical interventions. Two out of three operators showed an improvement in the operative time for performing the ovarian cystectomy, and the third operator showed a decrease in the time for performing interventions on the fallopian tubes. This threshold of 20 laparoscopic interventions may mark an improvement in the skills for this type of surgery. After learning the laparoscopic techniques and decreasing the operating time, an increase in the difficulty of the cases selected for laparoscopic intervention can be noted.

A major problem regarding the training of residents was represented by the lack of established learning protocols. Many times, the gynecologists who practiced in university hospitals were still dealing with their learning curve and thus there was a reduced opportunity for the residents to practice in the operating room. Other negative factors were represented by the reduced number of resident working hours or the small number of major laparoscopic interventions [17].

Acquiring skills in laparoscopic surgery, such as manipulating the camera and instruments, hands-eye coordination, and depth perception, is difficult and requires a long learning process. The possibility to practice repeatedly without endangering the safety of the patient is represented by medical simulators. Halstead’s classic surgical teaching principle “see one, do one, teach one” has lost value in front of new medical simulation techniques that can improve laparoscopic skills but without risk for patients [38]. Medical simulation is an effective way of learning laparoscopy. However, its implementation is difficult due to considerations related to money and time [39].

The limitation of the time given to teaching laparoscopy determined the need to develop some didactic methods for learning laparoscopy outside the operating room. Thus, several methods of practicing laparoscopic techniques appeared. They have been developed from simple training boxes to simulators that use virtual reality. A controversial practice is preparation on cadavers [40].

Traditionally, surgical techniques were acquired in the operating room and were dependent on a mentor. The effectiveness of this method was variable. Thus, the need to develop some modern training models for surgical procedures appeared. A study that analyzed 58 trails about training models in laparoscopy concluded that both virtual reality training and video training had the same results in the learning process. The two techniques represented valid learning methods, and their combination was recommended. The results are superior to those obtained by learning surgical techniques in the operating room. The results were contradictory in the case of practicing robotic technologies [41]. It was observed that the use of virtual reality training determined the reduction of operative time by 17–50% depending on the principles used for the exercise and the type of simulator. The implementation of training models in laparoscopy resulted in shortening the learning curve. Thus, it was concluded that the skills acquired through virtual reality training can be transferred to the operating room [11].

Another method of acquiring surgical skills is training at home. In this situation, each surgeon can adapt his practice style according to his needs. This method has become a feasible one for learning laparoscopy, and the self-evaluation process is important [42].

The difference between the use of 2D vs. 3D laparoscopy was also analyzed. In the case of using a box trainer, the number of errors was significantly higher among those who used the 2D technique compared to those who used the 3D technique. The time required to perform a laparoscopic hysterectomy was not significantly different between the two groups. It was concluded that 3D laparoscopy is useful for beginning surgeons and facilitates the learning process [43]. A study by Degirmenci et al. showed that in the case of 3D laparoscopy performed for complex urogynecological pathologies, a shorter operating time and a smaller amount of blood loss were found than in the case of 2D laparoscopy [44].

Single-site laparoendoscopic surgery (LESS) was developed and the surgical intervention is performed by using a single transumbilical multiport. In several studies, the feasibility and safety of this surgical technique in the treatment of benign gynecological diseases was confirmed. This presents a series of advantages compared to conventional laparoscopic surgery, such as a lower rate of postoperative complications, a faster recovery, and a lower need for postinterventional analgesia. This technique requires advanced laparoscopy skills, which makes it difficult to be accepted on a large scale. There are studies that have shown that between 30 and 55 interventions of this type are necessary for benign gynecological pathologies to master the technique [45].

Robotic surgery developed from the desire to perfect laparoscopic instruments and to obtain an image of increased quality. This seems to be developing as a separate field. The use of minimally invasive surgical techniques in the treatment of gynecological diseases is encouraged [46]. A meta-analysis concluded that mortality is similar between the laparoscopic and the robotic approaches [47].

## 5. Conclusions

The process of learning laparoscopy is laborious and difficult compared to learning classical surgery techniques. We identified a significant decrease in operating time after twenty laparoscopy interventions. After learning the laparoscopic techniques, there may be an increase in operative time by dealing with complicated surgical cases.

It is important to develop and improve new models of laparoscopy learning, from practicing at home and using box trainers to virtual reality training or video training.

## Figures and Tables

**Table 1:** The duration of surgery.

Variables	N	Duration (Mean ± Std Dev) (min)	*p*
Pathology			0.161
Tubal	56	109.14 ± 27.55	
Ovarian	103	115.50 ± 26.99	
Laparoscopic approach			0.786
Hasson	64	113.98 ± 28.81	
Veress	95	112.78 ± 26.34	
Number of trocars			0.809
2	2	130.00 ± 14.14	
3	92	112.29 ± 28.02	
4	64	114.03 ± 26.77	
5	1	120.00	
Days of hospitalization			0.405
3	26	119.62 ± 24.61	
4	96	110.88 ± 28.81	
5	35	114.14 ± 25.07	
6	2	130.00	
Operator			0.227
A	47	119.02 ± 25.13	
B	59	110.68 ± 29.07	
C	53	111.04 ± 26.77	

**Table 2:** Laparoscopic ovarian cystectomy and adnexectomy.

Type of Surgery	Operator A	Operator B	Operator C
Laparoscopic ovarian cystectomy			
N (%)	29 (61.7%)	27 (45.8%)	20 (37.7%)
Duration (mean ± std)	118.41 ± 23.96	114.00 ± 28.87	106.25 ± 22.23
Complications	1	-	1
Laparoscopic adnexectomy			
N (%)	4 (8.5%)	6 (10.2%)	10 (18.9%)
Duration (mean ± std)	120.00 ± 16.33	119.17 ± 39.04	129.00 ± 24.24
Complications	-	-	-

**Table 3:** Laparoscopic ovarian cystectomy.

Type of Surgery	N	Duration (Mean ± Std) (min)	*p*
Operator A			0.01
Phase I	17	127.65 ± 15.52	
Phase II	12	105.33 ± 28.16	
Operator B			0.01
Phase I	13	127.69 ± 23.05	
Phase II	14	101.29 ± 28.54	
Operator C			0.48
Phase I	5	100.00 ± 41.83	
Phase II	15	108.33 ± 12.34	

**Table 4:** Laparoscopic salpingectomy.

Type of Surgery	N	Duration (Mean ± Std)	*p*
Operator A			-
Phase I	0	-	
Phase II	7	122.86 ± 29.84	
Operator B			0.904
Phase I	6	101.67 ± 21.13	
Phase II	15	103.13 ± 26.16	
Operator C			0.000
Phase I	8	137.50 ± 21.87	
Phase II	11	91.82 ± 23.48	

## Data Availability

The data used in this study are available from the corresponding author and the authors can share the information if they are reasonable request.
